# Perilobar Nephroblastomatosis: Natural History and Management

**DOI:** 10.1155/2014/756819

**Published:** 2014-07-09

**Authors:** S. Stabouli, N. Printza, J. Dotis, A. Matis, D. Koliouskas, N. Gombakis, F. Papachristou

**Affiliations:** ^1^1st Department of Pediatrics, Aristotle University of Thessaloniki, Hippokration Hospital of Thessaloniki, 49 Kostantinoupoleos Street, 54642 Thessaloniki, Greece; ^2^Pediatric Oncology Clinic, Hippokration Hospital of Thessaloniki, 49 Kostantinoupoleos Street, 54642 Thessaloniki, Greece

## Abstract

Nephroblastomatosis (NB) has been considered as a precursor of Wilms tumor (WT). The natural history of NB seems to present significant variation as some lesions may regress spontaneously, while others may grow and expand or relapse and develop into WT later in childhood. Although, most investigators suggest adjutant chemotherapy, the effect and duration of treatment are not well established. Children with diffuse perilobar NB, Beckwith-Wiedemann syndrome, and hemihypertrophy seem to particularly benefit from treatment. We discuss our experience on two cases of NB and we review the literature for the management of this rare condition.

## 1. Introduction

Nephroblastomatosis (NB) defines the presence of diffuse or multiple nephrogenic rests (NRs). NRs are clusters of embryonic metanephric cells, which normally disappear after 36 weeks of gestational age. These lesions have been considered as precursors of Wilms tumor (WT). They can be present in about 1% of unselected infant kidneys at postmortem biopsies, while they are found in about 40% of kidneys with unilateral WTs and in nearly 100% of kidneys with bilateral WTs [1]. NB has also significant implications for the prognosis of pediatric patients with WTs, as its presence in the nontumoral part of the kidney may favor subsequent relapse of WTs [2].

NB can occur in any age, but it is most frequent in infants. NB in about of 40% of cases is bilateral, while unilateral presentation may be implicated with the presence of microscopic NRs on the contralateral kidney with increased risk of WT development. Limited publications have assessed the clinical course and the effect of management decisions on the outcome of children with NB. Most available data derive from small number of cases. In the current paper we discuss our experience on two cases of perilobar NB (PLNB) presented in our department with an interval of 20 years and we review challenging issues for the management of this rare condition.

## 2. Case Presentation

**Figure 1:**
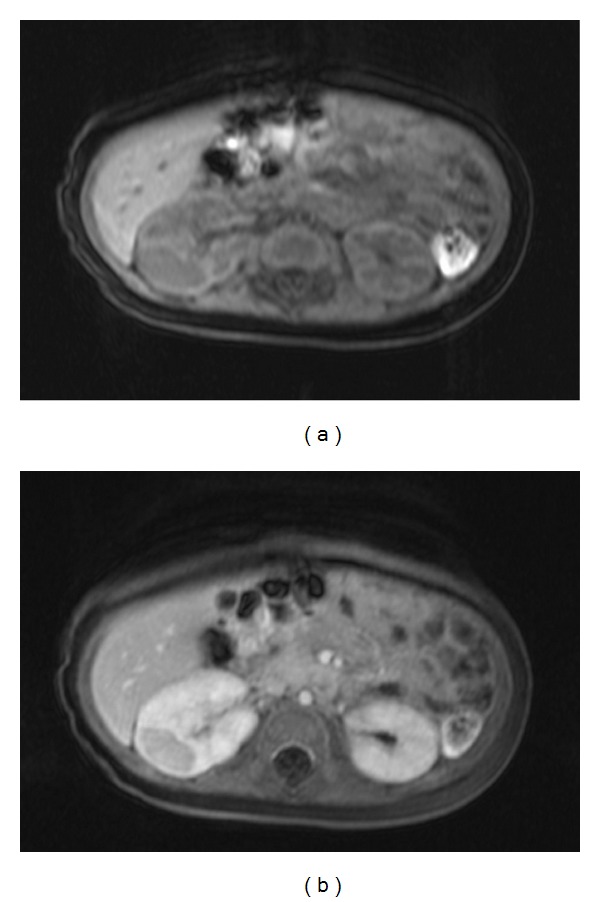
Noncontrast (a) and contrast enhanced (b) T1 weighted MR images show a large hypointense cortical mass at the right kidney and multiple smaller foci in both kidneys.

A 3.5-months-old girl was admitted to our department with right-sided hemihypertrophy. Screening with abdominal ultrasonography showed an enlarged right kidney with a large hypoechoic region presenting no corticomedullary differentiation as well as multifocal hypoechoic parenchymal foci bilaterally in both kidneys, suggesting PLNB. Magnetic Resonance Imaging (MRI) revealed multiple hypodense and nonenhancing cortical masses at both kidneys; the largest with a diameter of 2.65 cm was localized at the enlarged right kidney and presented reduced diffusion and faint enhancing tissue at periphery (Figures 1(a) and 1(b)). As all lesions were homogeneous without enhancement after contrast administration and a lenticular shape the diagnosis of PLNB was further suggested by the MRI findings. Spherical shape, heterogeneous, and enhancing nodules that would be suspicious for a WT were not present in the MRI. A second abdominal ultrasonography 2 months later showed enlargement of the already existing and new foci of NB bilaterally.

**Figure 2:**
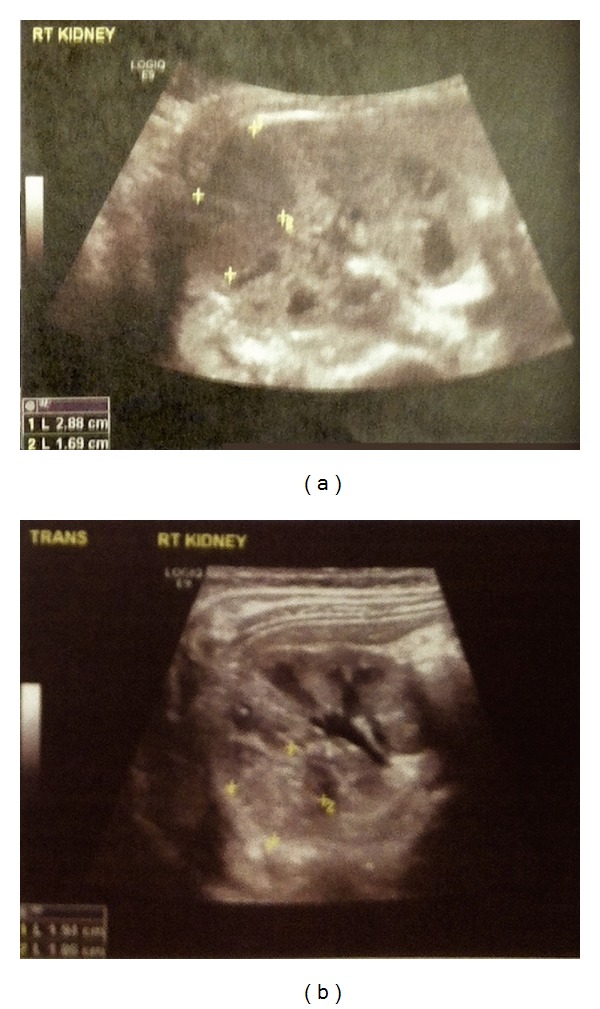
(a) Abdominal ultrasound showing an enlarged right kidney with a large hypoechoic region with no corticomedullary differentiation before chemotherapy treatment; (b) decrease of right kidney large hypoechoic lesion dimensions after 4 months of treatment.

Some years ago we presented the case of a 23-month-old boy, who did not received any treatment for the initial diagnosis of right NB and developed WTs 24 and 42 months later at the left and the right kidney, respectively, despite regression of initial lesions of NB [3]. Review of the literature on the management of NB revealed one large retrospective study and several case reports describing in most cases adverse outcome in nontreated patients. Thus, our female patient initiated chemotherapy according to SIOP Wilms Tumor/2001 protocol and received vincristine and actinomycin D for 4 weeks. Abdominal ultrasonography at 4 weeks showed decrease of lesion's size (shrinkage of the large right kidney mass volume from 7,56 cm^3^ to 3,26 cm^3^) and the patient received further cycles of vincristine and actinomycin D every 14 days for the next 3 months. Follow-up ultrasound at 4 months of treatment showed additional decrease of lesions dimensions (Figure 2). However, the follow-up period is currently too short to allow us to determine the response to treatment with confidence.

## 3. Discussion

In 1990 Beckwith et al. proposed the classification for NB into four categories: the perilobar (PLNB), intralobar (ILNB), combined, and universal [1]. All four categories have been associated with WT, PLNB with synchronous bilateral WTs, and ILNB with metachronous contralateral WTs. NRs and NB have been reported to have an increased frequency in several syndromes, including Beckwith-Wiedemann syndrome, hemihypertrophy, Perlman syndrome, and trisomy 18 [1, 4]. Nodular appearance may be more frequent in association with the presence of the above syndromes although diffuse pattern has also been reported [4, 5]. Pediatric patients with Beckwith-Wiedemann syndrome and idiopathic hemihypertrophy also have an increased risk, reported about 4%–10%, for developing embryonic tumors [4]. The clinical course of PLNB presents large variation; some lesions may grow and expand or decrease or fade and relapse later in childhood. The risk of developing one or more WTs during the natural history of disease is increased, especially in cases with diffuse hyperplastic PLNB (DHPLNB) [5]. DHPLNB presents as massive kidney enlargement due to thick ride of nephroblastic tissue. DHPLNB has also been associated with increased incidence of anaplastic WTs [5, 6].

As nephroblastomatosis is a preneoplastic condition, administration of chemotherapy could be considered under the concept of decreasing the volume of lesions and reducing the number of cells with malignant potential and subsequently the risk of malignant transformation [5, 7]. Treatment of NB with vincristine and actinomycin D is currently recommended as for stage 1 WT. However, chemotherapy may not be effective or prevent malignant transformation. Moreover, there are currently limited data in the literature to assess this issue with confidence.

Observation and close follow-up may be an option although epidemiologic evidence may not favor such decision. The main arguments in favor of nontreatment are the possible side effects of chemotherapy applied for nonmalignant condition, which has usually a favorable prognosis even when WT is developed. Moreover, chemotherapy may enhance the selection of resistant tumors [5, 7]. There are sporadic reported cases with spontaneous resolution of NB without treatment. However, the risk of developing WT seems to persist even years after initial diagnosis. Our male patient described above, who did not receive any treatment, presented spontaneous resolution of left kidney NB but developed new foci of NB and metachronous WT at the right kidney [3].

Forty-one individual cases have been published in the literature since 1978, of which 26 were after the classification from Beckwith et al. (Table 1) [7–25]. Observation of reported cases provides some evidence of the natural history of disease, but could not result in generalized conclusions about treatment decisions. Of nine with PLNB patients who received chemotherapy as initial treatment, seven developed WT at a mean of 29.9 months from diagnosis. Only one patient presented anaplastic pathology. All patients had a favorable outcome. Three patients did not receive any treatment; one of those suffering from PLNB developed WT, while the others have been followed up for a too short period. Ten cases of newborns with NB detected in postmortem biopsies were reported. The majority of these cases were associated with congenital abnormalities and died within the first days of life. In two cases, in which renal failure was a predominant feature, NB was found at biopsies performed after native nephrectomies during renal transplantation.

One large series of 52 patients provides data on patients with long-term survival of HPLNB [5]. The patients were followed up for at least 5 years. The lesions were bilateral in 49/52 cases, 45/52 had DHPLNB, and 8/52 patients had features of Beckwith-Wiedemann or other syndromes. Only three patients were observed without receiving chemotherapy at diagnosis. All three developed WT subsequently at 4 and 10 months later. Similar was the clinical course in our first case, as described earlier. Of the remaining 49 patients who received chemotherapy all presented an initial decrease in lesions volume. However, 55% of those that received only chemotherapy developed WT, while among patients who were treated with nephrectomy and chemotherapy 19% (three patients) developed WT. Chemotherapy seems to delay the occurrence of WT in patients with HPLNB. In the study by Perlman et al., the mean time from initial diagnosis of HPRNB to the appearance of WT was 35 months in treated pediatric patients (range of 12–60 months) compared to mean of 6.5 months in those who did not receive treatment. Similarly in cases in Table 1, WT developed in shorter time period if chemotherapy was administrated (35 months versus 12 months in the nontreated patients). Even if the patient develops WT during treatment the delay of appearance may allow nephron-sparing approaches.

Another interesting issue concerning the clinical course of PLNB is that the speed of the response to chemotherapy, which may suggest the duration of chemotherapy, presented significant variation among reported cases. In many cases prolonged chemotherapy is required to achieve regression of disease [5]. These observations may suggest that the duration of chemotherapy in children with PLNB needs to be continuously assessed during follow-up and treatment. DHPLNB may represent increased burden of disease. Moreover, in the cohort described by Perlman et al., children who presented relapses with new lesions during chemotherapy and children with genetic syndromes had an increased risk for WT. These children may need prolonged treatment. In the case of our female patient the cluster of unfavorable prognostic factors including hemihypertrophy and transient initial response to treatment reinforce the decision for chemotherapeutic treatment. Genetic analysis for mutations in WT1, WT2, and WTX genes may further guide the duration and the intensity of chemotherapeutic schemes. An ongoing trial on the effect of chemotherapy in preserving renal units in children with DHPLNB and preventing WT development may give guidance for the management of disease [6]. Patients will initially receive vincristine and * *actinomycin D and maybe partial nephrectomy after initial chemotherapy, especially if there is no response or if there is progression of disease or development of new lesions during therapy.

In conclusion, chemotherapy maybe the optimal treatment decision for pediatric patients with PLNB. Current evidence favor the individualization of treatment and close follow-up of the children with PLNB as suggested for individuals with increased risk for WT [6]. Patients should be followed up by imaging at a maximum interval of 3 months for a minimum of 7 years, as early detection of a WT may be critical for patient and kidney survival.

## Figures and Tables

**Table 1:** Cases with initial diagnosis of NB (NB) without synchronous WT published since 1990.

Case	Reference	Age at diagnosis	Sex	Diagnosis	Clinical presentation at diagnosis	Congenital defects	Biopsy for NB	Ch for NB/ duration	Response to initial Ch	Srg For NB	Ra For NB	Development of WT	Outcome/ follow-up since initial diagnosis
1	Gaulier et al., Pediatr Pathol., 1993 [8]	Newborn		Unilateral universal NB	Cystic renal process discovered prenatally	Yes	Yes	No	—	Yes	No	No	Alive/1 yr

2	Regalado et al., Pediatr Pathol.,1994 [9]	Newborn	M	Bilateral universal NB	Potter's-like facies, hypoplastic lungs, ascites, and bilateral nephromegaly	Yes	Yes (postmortem)	No	—	No	No	No	Dead at age of 21 h

3	Verloes et al., Clin Genet., 1995 [10]	Newborn		Bilateral NB	Fetal overgrowth, macroglossia, and ambiguous genitalia	Yes/atypical Simpson-Golabi-Behmel and Beckwith-Wiedemann S	Yes (postmortem)	No	—	No	No	No	Dead at age of 2 days

4	Regalado et al., Pediatr Pathol Lab. Med., 1996 [11]	Newborn	M	Bilateral universal NB	Prenatally diagnosed nephromegaly and renal failure	No	Yes (postmortem)	NO	—	No	No	No	DOD at age of 3.5 mo

5	Henneveld et al., Am J Med Genet., 1999 [12]	8 mo	F	Unilateral NB	Nephromegaly FTH and other features of Perlman S	Yes/Perlman S	Yes (postmortem)	NO	—	No	No	No	Dead

6	Spranger et al., J Clin Dysmorphol., 2001 [13]	8 mo	M	Peri- and intralobar NB	Macrocephaly and short trunk	Yes/Ischiospinal dysostosis with rib gaps	Yes	NO	—	No	No	No	Alive/2 mo

7	Prasil et al., Med Pediatr Oncol., 2000 [7]	15 mo	M	Bilateral HPLN	Abdominal mass	No	Yes	5 course VCR-AMD/20 wks	Partial regression	No	No	Yes/5 yrs	Alive/5.5 yrs

8	Prasil et al., Med Pediatr Oncol., 2000 [7]	13 mo	M	Unilateral HPLN	Abdominal mass	No	Yes	3 course VCR-AMD/24 wks	Partial regression	No	No	Yes/28 mo	Alive/3.5 yrs

9	Prasil et al., Med Pediatr Oncol., 2000 [7]	3 yrs	F	Bilateral HPLN	Abdominal mass	No	Yes	2 course VCR-AMD/20 wks	Partial regression	NO	NO	Yes, multifocal with anaplasia/18 mo	NED/4 yrs

10	Günther et al., Pediatr Radiol., 2004 [14]	2 yrs	F	Bilateral DHPLN	NR	NR	No	NR	—	NO	NO	Yes/12 mo	NR

11	Cozzi et al., J Urol., 2004 [15]	12 mo	F	Bilateral HPLN	Abdominal mass	No	No	2-drug/	—	NO	NO	Yes/4 wks	NED/6 yrs

12	Cozzi et al., J Urol., 2004 [15]	13 mo	F	Unilateral HPLN	Abdominal mass/pain	No	No	2-drug/10 wks	Complete regression	NO	NO	Yes/14 wks	NED/32 mo

13	Hu et al., Nephrol Dial Transplant., 2004 [16]	21 mo	M	Bilateral NB	Hypoplastic genitalia, glomerulopathy, and renal failure	Yes/atypical Denys-Drash S and mutation of WT1 gene	Yes (nephrectomy at time of TN)	No	—	NO	NO	NO	NR

14	Hu et al., Nephrol Dial Transplant., 2004 [16]	6 yrs	M	Bilateral NB	Pseudohermaphroditism, glomerulopathy, and renal failure	Yes/atypical Denys-drash S and mutation of WT1 gene	Yes (nephrectomy at time of TN)	No	—	NO	NO	NO	NR

15	Christiansen et al., Pediatr Dev Pathol., 2005 [17]	Newborn	F	Bilateral DHPLN	Congenital heart disease, anddiaphragmatic hernia	Yes/mosaic duplication 1(q11q44)	Yes (postmortem)	No	—	NO	NO	NO	Dead at first day of life

16	Machmouchi et al., Pediatr Nephrol., 2005 [18]	8 mo	F	Bilateral HPLN	Abdominal distention/respiratory distress/macroscopic hematuria	No	Yes	VRC-AMD-DX/24 wks	Partial regression	NO	NO	NO	NED/1 yr

17 (sibl. of 18)	Gonzales et al., Am J Med Genet., 2005 [19]	Newborn	M	Bilateral NB	Lumbosacral meningocele, large cystic and dysplastic kidneys, and oligohydramnios	Yes/diaphanospondylodysostosis	Yes (postmortem)	No	—	NO	NO	No	Dead at first day of life

18 (sibl. of 17)	Gonzales et al., Am J Med Genet., 2005 [19]	Newborn	F	Bilateral NB	Oligohydramnios and cystic kidneys	Yes/diaphanospondylodysostosis	Yes (postmortem)	No	—	No	No	No	Dead at first day of life

19	Traub et al., Virchows Arch., 2006 [20]	Fetus 24 weeks	M	Bilateral diffuse peri- and intralobar NB		Yes/trisomy 13 and loss of WT1 expression	Yes (postmortem)	No	—	No	No	No	Dead at birth

20	Witt et al., J Pediatr Hematol Oncol., 2009 [21]	9 mo	F	Bilateral DHPLN	Abdominal distention/respiratory distress/acquired von Willebrand disease	Yes/hip dysplasia	Yes	VCR-AMD/122 wks 13-*cis* retinoic acid/9 wks VCR-AMD-DX/NR	Partial regression	No	No	Yes/31.5 mo	Alive/3.6 yrs

21	Vicens et al., Pediatr Dev Pathol., 2009 [22]	1 yr	M	Unilateral DHPLN	Abdominal mass	No	Yes	VCR-AMD/4 wks	Partial regression	Yes	No	No	NR

22 (sibl. of 22)	Katzman et al., Pediatr Dev Pathol., 2009 [23]	Newborn	F	Combined NB	Prenatally diagnosed nephromegaly	No	Yes (postmortem)	No	—	No	No	No	DOD at 6 day of life

23 (sibl. of 21)	Katzman et al., Pediatr Dev Pathol., 2009 [23]	Newborn	M	Intralobar universal NB	Prenatally diagnosed nephromegaly	No	Yes (postmortem)	No	—	No	No	No	DOD at 10th day of life

24	Borny et al., JBR-BTR., 2009 [24]	12 mo	F	Multifocal PLN	NR	Yes/Beckwith-Wiedemann S	NR	NR	NR	NR	NR	NR	NR

25	Sethi et al., Radiographics., 2010 [25]	6 mo	F	Bilateral DHPLN	Abdominal mass	No	No	No	—	No	No	Yes/12 mo	Alive/NR

26	Rauth et al., J Pediatr Surg., 2011 [26]	10 mo	F	Bilateral DHPLN	Urinary infection	No	No	2 courses of VCR-AMD/18 wks and 24 wks	Partial regression	No	No	Yes/3.5 yrs	NR

Abbreviations: NB: nephroblastomatosis, Ch: chemotherapy, Srg: surgery, Ra: radiation, WT: Wilms tumor, f: female, m: male, sibl: sibling, HPRN: hyperplastic perilobar NB, DHPRN: diffuse hyperplastic perilobar NB, VRC: Vincristine, AMD: dactinomycin, DX: doxorubicin, NR: not reported, DOD: dead of disease, NED: no evidence of disease, TN: transplantation.
